# Prevalence of diabetes among Han, Manchu and Korean ethnicities in the Mudanjiang area of China: a cross-sectional survey

**DOI:** 10.1186/1471-2458-12-23

**Published:** 2012-01-10

**Authors:** Yan Feng, Yumei Yang, Xuesong Ma, Kaiting Chen, Nannan Wu, Dongmei Wang, Pengjie Li, Minnan Wang, Qiang Li, Jinchao Zhang

**Affiliations:** 1 Department of Endocrinology and Metabolism, The Second Affiliated Hospital, Harbin Medical University, Harbin 150086, China; 2 Department of Endocrinology and Metabolism, The Second Hospital of Mudanjiang City, Mudanjiang 157000, China

**Keywords:** Type 2 diabetes mellitus, Ethnic differences, Risk factors

## Abstract

**Background:**

Rapid socioeconomic development resulting in changing lifestyles and life expectancy appears to be accompanied by an increasing prevalence of type 2 diabetes. Genetic predisposition related to ethnicity is a major determinant of diabetes risk. This study investigates the prevalences of diabetes and prediabetes in different ethnic populations residing in the Mudanjiang area located in the northeast of China.

**Methods:**

A cross-sectional survey was carried out among Han, Manchu and Korean Chinese aged 20 years or older. Diabetes and prediabetes were diagnosed using standard oral glucose tolerance tests.

**Results:**

The prevalence of diabetes in Manchu (8.39%) and Korean Chinese (9.42%) was significantly lower than that in Han (12.10%). The prevalence of prediabetes was 18.96%, 19.36% and 20.47% in Han, Manchu and Korean populations, respectively. Korean Chinese had a lower prevalence of isolated impaired fasting glucose and higher prevalence of isolated impaired glucose tolerance than the other two ethnic groups. Most patients with diabetes, especially ethnic minority patients, were undiagnosed. A multiple logistic regression analysis showed that age, family history of diabetes, control of diet, self-monitoring of weight, central obesity, increased heart rate, hypertension, elevated plasma triglyceride level, elevated plasma low-density lipoprotein cholesterol, and Han ethnicity were significantly associated with an increased risk of diabetes. Further, Manchu Chinese were found to have the lowest risk of diabetes.

**Conclusions:**

Our study indicates that diabetes is a major public health problem in the Mudanjiang area of China. Ethnicity plays a role in the different prevalences of diabetes and prediabetes among the three ethnic groups. Diabetes is less prevalent among Manchu Chinese compared with Han and Korean Chinese.

## Background

Currently, over 180 million people worldwide have diabetes, and this number is expected to double by 2030 [1]. Amid the rapid socioeconomic development resulting in changing lifestyles and life expectancy, the prevalence of diabetes in China is high and is still increasing [2-4]. Mudanjiang is located in the northeast of China, has a temperate monsoon climate, and has a heterogeneous population comprising several ethnic groups that differ in language, socioeconomic status, dietary habits, and elapsed time since immigration, which are characteristics that are different from those observed in other areas of China. Han Chinese are the main ethnic group, and Manchu and Korean Chinese are the main ethnic minorities in this area. In addition to the genetic risk associated with a family history of diabetes, a genetic predisposition related to ethnicity is a major determinant of diabetes risk. This has been clearly demonstrated in Amsterdam and Taiwan, where the prevalences of diabetes in different ethnic groups were significantly different [5,6]. So far, there is no information on the prevalence of diabetes and prediabetes among Han, Manchu and Korean individuals in the Mudanjiang area. Therefore accurate information on the prevalence of diabetes among Han, Manchu and Korean Chinese people living in the Mudanjiang area is needed. It is unknown whether differences in demographic variables, including ethnic background, play a role in the prevalence of diabetes among the three ethnic groups. More information on the differences in determinants of diabetes among ethnic groups can help to define specific strategies for the prevention of diabetes.

To address these major gaps in knowledge, we investigated the prevalences of diabetes and prediabetes in Han, Manchu and Korean populations living in the Mudanjiang area of China.

## Methods

### Study participants

A cross-sectional survey was conducted in the Mudanjiang area. Eligible participants were Han, Manchu and Korean Chinese people aged 20 years or older, who had lived in their current residence for 5 years or longer. To select a representative sample, the sampling process was stratified according to economic development status (city, town and village). At first, cities and counties were non-randomly selected from the Mudanjiang area. Then, four urban street districts, four town districts and eight rural villages were randomly selected from the cities and counties. All eligible residents in the selected districts were invited to participate in the study.

A total of 4400 eligible residents were invited to participate in the survey, of which 3984 (1782 men and 2202 women) completed the study.

The study protocol was approved by the institutional review board of participating institutions and the Second Affiliated Hospital Ethics Committee of Harbin Medical University. Written informed consent was obtained from each participant before data collection.

### Data collection

Interviews were conducted by trained staff. A questionnaire was used to obtain information on demographic characteristics, personal and family medical history, and lifestyle risk factors. The participants' medical records were reviewed, including the diagnosis and treatment of diabetes. Cigarette smoking was defined as having smoked at least 100 cigarettes in one's lifetime. Information was obtained on the amount and type of alcohol that was consumed during the previous year, and alcohol drinking was defined as the consumption of at least 30 g of alcohol per week for 1 year or more. Regular physical exercise was defined as exercising moderately or strenuously for at least 30 min per day, 3 days a week. Educational level and income were also recorded. Educational level was classified by senior high school or above and junior high school or below. Economic level was regarded as low (less than 10 000 YUAN per annum) or not low (10 000 YUAN or more per annum). Blood pressure, heart rate, body weight, height, and waist circumference were measured with the use of standard methods, as described previously [4]. Body mass index (BMI) was calculated using the formula BMI = mass (kg)/(height [m])^2 ^and waist-to-hip ratio (WHR) was calculated by dividing waist circumference (cm) by hip circumference (cm). Overweight was defined as a body mass index between 25.0 and 29.9, and obesity as a body mass index of 30.0 or more [7]. Central obesity was defined as a waist circumference of 90 cm or more in men and 80 cm or more in women [7].

### Oral glucose tolerance test and lipids measurements

Before the oral glucose-tolerance test, Participants were asked to maintain their usual physical activity and diet for at least 3 days. After an overnight fasting of at least 10 h, a venous blood sample was collected for the measurement of plasma glucose. Participants with no history of diabetes performed a standard 75-g oral glucose tolerance test (OGTT). Participants who had been diagnosed with diabetes were given a steamed bun that contained approximately 80 g of complex carbohydrates. Blood samples were drawn at 0 and 120 min after the glucose or carbohydrate load to measure plasma glucose concentrations.

Plasma glucose and blood lipid profiles were examined using commercially available diagnostic reagents at the clinical biochemical laboratories in the Second Affiliated Hospital of Harbin Medical University. Plasma glucose was measured using an enzymatic colorimetric method with glucose oxidase. Total cholesterol (TC, normal values 1.80-5.17 mmol/l) was assayed using the enzymatic colorimetric method with cholesterol esterase and cholesterol oxidase. High-density lipoprotein cholesterol (HDL-C, normal values 1.04-1.70 mmol/l) was measured after precipitation of the apolipoprotein B containing lipoproteins with phosphotungstic acid. Triglycerides (TGs, normal values 0.56-1.70 mmol/l) were assayed using enzymatic colorimetric assay with glycerol phosphate oxidase. Low-density lipoprotein cholesterol (LDL-C, normal value 0.45-3.15 mmol/l) was measured using the enzymatic colorimetric method.

### Study outcome definitions

The 1999 World Health Organization (WHO) diagnostic criteria were used for the diagnosis of diabetes. Participants were classified according to the results of plasma glucose testing as follows:

$$\begin{array}{c} \text{normal}\;\text{glucose}\;\text{tolerance}\left(\text{NGT}\right)-(\text{fasting}\;\text{glucose}\;[\text{FG}]\;<\;6.1\;\text{mmol}∕\text{L},2-\text{hpostprandial}\;\text{glucose}\\ \left[\text{PPG}\right]<\;7.8\;\text{mmol}∕\text{L},\;\text{and}\;\text{no}\;\text{diagnosis}\;\text{of}\;\text{diabetes}); \end{array}$$

isolatedimpairedfastingglucose(IFG)-(7.0mmol∕l>FG≥6.1mmol∕l,and2-hPPG<7.8mmol∕l);

$$\begin{array}{c} \text{isolated}\;\text{impaired}\;\text{glucose}\;\text{tolerance}\left(\text{IGT}\right)-(\text{FG}<6.1\;\text{mmol}∕\text{l},\text{and}\\ 11.1\;\text{mmol}∕\text{l}>2-\text{hour}\;\text{PPG}\geq 7.8\;\text{mmol}∕\text{l}); \end{array}$$

$$\begin{array}{c} \text{combined}\;\text{impaired}\;\text{fasting}\;\text{glucose}\;\text{and}\;\text{impaired}\;\text{glucose}\;\text{tolerance}\;\left(\text{combined}\;\text{IFG}\;+\;\text{IGT}\right)-\\ \left(7.0\;\text{mmol}∕\text{l}\;>\;\text{FG}\geq 6.1\;\text{mmol}∕\text{l},\text{and}\;11.1\;\text{mmol}∕\text{l}>2-\text{hour}\;\text{PPG}\geq 7.8\;\text{mmol}∕\text{l}\right); \end{array}$$

$$\text{diabetes}-\left(\text{FG}\geq 7.0\;\text{mmol}∕\text{l},2-\text{hour}\;\text{PPG}\geq 11.1\;\text{mmol}∕\text{l},\text{or}\;\text{both}\right).$$

Previously diagnosed diabetes was identified by a positive response from the participant to the question, "Has a doctor ever told you that you have diabetes?" Prediabetes was defined as IFG, IGT or both. Diabetes included both previously diagnosed diabetes and previously undiagnosed diabetes.

### Statistical analysis

The purpose of the present study was to compare the prevalence of diabetes and prediabetes among three ethnic groups in the population residing in the Mudanjiang area. Continuous data are expressed as means ± standard deviations (SD). Categorical data are expressed as percentages. *t*-tests, one way analysis of variance, or nonparametric tests (chi-square) were used to evaluate the differences in variables and outcomes among different groups, as appropriate. One-way analysis of variance (ANOVA) was used to adjust for the effects of age and gender on the various risk factors. To provide information on the prevalence of diabetes in the Mudanjiang area, we corrected for over sampling and non-response by weighting our study sample by the age and gender distribution of the Han, Manchu and Korean populations living in the Mudanjiang area. Multiple logistic regression was used to estimate the odds associated with diabetes and prediabetes for selected risk factors while controlling for potential confounding factors. The Han ethnic group was used as a reference in all analyses. A two-tailed *p*-value of < 0.05 indicated statistical significance. Statistical analyses were performed with the SAS software package 9.1.

## Results

### Characteristics of participants by plasma glucose categories and ethnicity

The demographic and clinical characteristics of participants by plasma glucose categories and ethnicity are summarized in Additional file 1. The mean ages of participants with prediabetes and diabetes were comparable among the three ethnic populations. More Han participants with diabetes reported a family history of diabetes than participants in the other two ethnic groups (*p *< 0.01). The rates of cigarette smoking, consumption of alcohol, and physical activity, and the economic level of participants with diabetes or prediabetes varied among the three ethnic populations. Korean Chinese participants with diabetes had less glycemic control than the other ethnic populations with respect to FG and 2-h PPG. No clinically relevant differences were observed among the three ethnic groups with respect to BMI, physical examination and lipid profile.

### Prevalence of diabetes and prediabetes among Han, Manchu and Korean people in the Mudanjiang area of China

The prevalence of diabetes was summarized by previously diagnosed diabetes and previously undiagnosed diabetes. Prediabetes was classified as isolated IFG, isolated IGT or combined IFG + IGT (Table 1). The prevalence of diabetes in Manchu (8.39%) and Korean Chinese (9.42%) in the Mudanjiang area was significantly lower than that in Han Chinese (12.10%). The prevalence of prediabetes was 18.96%, 19.36% and 20.47% in ethnic Han, Manchu and Korean populations, respectively. It is worthy of note that Korean Chinese had a lower prevalence of isolated IFG and higher prevalence of isolated IGT than the other two ethnic groups (Table 1).

**Table 1:** Prevalences of diabetes and prediabetes among Han, Manchu and Korean populations in the Mudanjiang area of China

		Isolated Impaired Fasting Glucose (%)	Isolated Impaired Glucose Tolerance (%)	Combined Impaired Fasting Glucose and Impaired Glucose Tolerance (%)	Previously Undiagnosed Diabetes (%)	Previously Diagnosed Diabetes (%)	*P *value
total	Han (n = 2695)	123(4.56)	246(9.13)	142(5.27)	228(8.46)	98(3.64)	
	Manchu (n = 620)	28(4.52)	68(10.97)	24(3.87)	46(7.42)	6(0.97)	
	Korean (n = 669)	17(2.54)	90(13.45)	30(4.48)	57(8.52)	6(0.90)	*P *< 0.0001
male	Han (n = 1237)	63(5.09)	104(8.41)	67(5.42)	113(9.13)	50(4.04)	
	Manchu (n = 264)	14(5.30)	32(12.12)	7(2.65)	23(8.72)	1(0.38)	
	Korean (n = 281)	9(3.20)	36(12.81)	12(4.27)	23(8.19)	1(0.36)	*P *= 0.0008
female	Han (n = 1458)	60(4.12)	142(9.74)	75(5.14)	115(7.89)	48(3.29)	
	Manchu (n = 356)	14(3.93)	36(10.11)	17(4.78)	23(6.46)	5(1.41)	
	Korean (n = 388)	8(2.06)	54(13.92)	18(4.64)	34(8.76)	5(1.29)	*P *= 0.0580

*p *value was calculated by chi-square tests

The prevalence of previously undiagnosed diabetes among the three ethnic groups was significantly higher than that of previously diagnosed diabetes. Previously undiagnosed diabetes accounted for 69.94% of the total diabetes in the Han population, 88.46% in the Manchu population and 90.48% in the Korean Chinese population.

The prevalences of prediabetes and were 18.92%, 20.70% and 20.28% for men and 19.00%, 18.82% and 20.62% for women in the Han, Manchu and Korean Chinese populations, respectively. The prevalences of diabetes were 13.17%, 9.10% and 8.55% for men and 11.18%, 7.87% and 10.05% for women in the Han, Manchu and Korean Chinese populations, respectively. The prevalence of diabetes was higher among men than women in Han and Manchu populations, but the reverse in the Korean Chinese population.

### Age-specific prevalences of diabetes and prediabetes among adults aged 20 years or older in the Mudanjiang area

**Figure 1:**
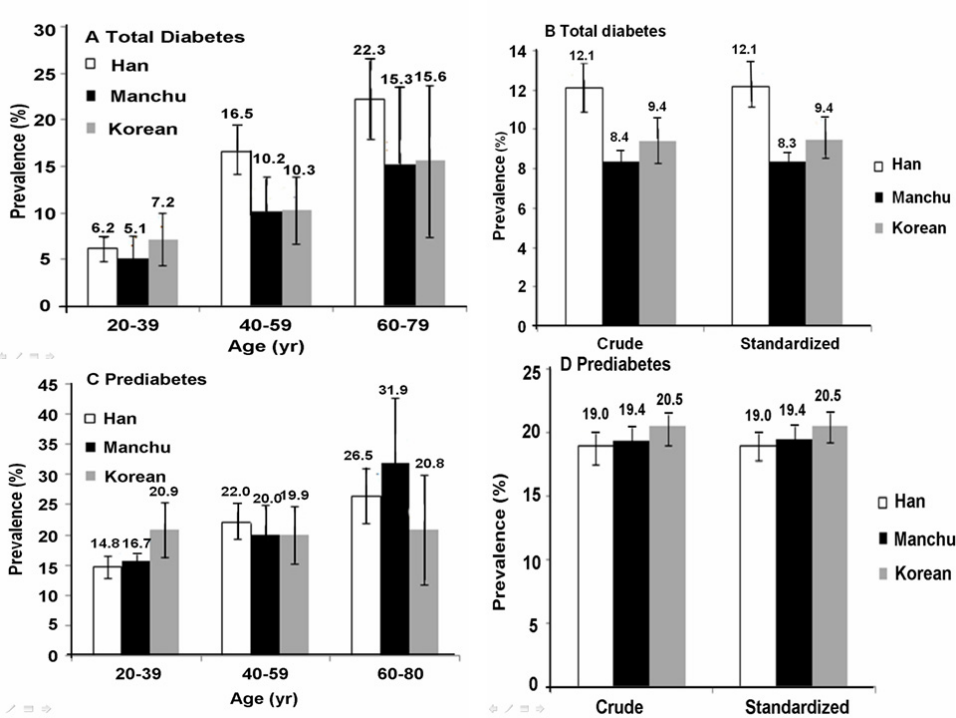
**Age-specific prevalences of diabetes and prediabetes among adults (aged 20 years or older) in the Mudanjiang area**. The prevalences of total diabetes (**A**) and prediabetes (**C**) among Han, Manchu and Korean Chinese are shown by age. The crude and age-and gender-standardized prevalences of total diabetes and prediabetes among Han, Manchu and Korean Chinese are shown in B and D, respectively. I bars indicate 95% confidence intervals.

The age-specific prevalences of diabetes and prediabetes increased with age (Figure 1A, C). The age-standardized prevalence of diabetes was 12.1% in the Han population and 8.3% in the Manchu population (*p *= 0.0145) (Figure 1B). The age-standardized prevalences of prediabetes were comparable among the three ethnic groups (*P *= 0.6664) (Figure 1D).

The prevalence of diabetes among people aged 20-40 years was similar in each of the ethnic groups. However, the prevalence of diabetes among people aged 40-60 years and 60-80 years was significantly higher in Han Chinese than in Manchu and Korean Chinese populations (Figure 1A).

The prevalence of prediabetes in the Han and Manchu populations increased with age. However, in the Korean Chinese population, the prevalence of prediabetes was quite even among the different age groups. In people aged 20-40 years, the highest prevalence of prediabetes was observed among Korean Chinese (*p *< 0.05), while in the 40-60 year old group and 60-80 year old group, the Han and Manchu populations had a higher prevalence of prediabetes, respectively (both *p *< 0.05) (Figure 1C)

### Multivariate risk assessment

In the multiple logistic regression model, age, family history of diabetes, control of diet, self-monitoring of weight, central obesity, increased heart rate, hypertension, elevated plasma triglyceride level, elevated plasma LDL cholesterol and Han ethnicity were all significantly associated with an increased risk of diabetes (Table 2). The Manchu Chinese population had a lower risk of diabetes. In addition, age, family history of diabetes, control of diet, self-monitoring of weight, central obesity, increased heart rate, hypertension, elevated plasma triglyceride level, and elevated plasma LDL cholesterol were significantly associated with an increased risk of prediabetes. Ethnicity was not an independent risk factor for prediabetes.

**Table 2:** Multivariate-adjusted odds ratios for diabetes and prediabetes

Variable	Total Diabetes (n = 441)		Prediabetes (n = 768)	
	
	Odds Ratio(95% CI)	*P *Value	Odds Ratio(95% CI)	*P *Value
Age, per increase of 10 years old	2.124 (1.838-2.454)	< 0.0001	1.519 (1.352-1.706)	< 0.0001
Family history	1.119 (1.084-1.154)	< 0.0001	1.172 (0.981-1.399)	0.0803
Self-monitor of Weight	1.057 (0.977-1.144)	0.1654	1.073 (1.009-1.141)	0.0243
Control of Diet	1.078 (1.001-1.161)	0.0458	1.094 (1.032-1.159)	0.0024
Resting Heart rate	1.620 (1.350-1.943)	< 0.0001	1.191 (1.030-1.376)	0.0182
Manchu vs Han	0.691 (0.500-0.955)	0.0251	1.017 (0.808-1.281)	0.8827
Korean vs Han	0.805 (0.596-1.086)	0.1560	1.082 (0.869-1.348)	0.4790
Central Obesity	1.371 (1.090-1.725)	0.0070	1.668 (1.396-1.992)	< 0.0001
Hypertension	1.982 (1.588-2.473)	< 0.0001	1.400 (1.166-1.681)	0.0003
LDL-C, per increase of 0.56 mmol/L (50 mg/dl)	1.320 (1.142-1.525)	0.0002	1.189 (1.058-1.337)	0.0037
TG, per increase of 0.56 mmol/L (50 mg/dl)	1.119(1.084-1.154)	< 0.0001	1.086(1.055, 1.118)	< 0.0001

Odds ratios were calculated using multivariate logistic regression models. All covariates listed were simultaneously included in the model. Cigarette smoking, alcohol consumption, level of leisure-time physical activity, serum cholesterol levels, educational level, and level of economic development were not significantly associated with the risk of diabetes and were not included in the final model. In this study, the data of BMI and WR have significant correlation, so we selected central obesity in the final model. Central obesity was defined as a waist circumference of 90 cm or more in men and 80 cm or more in women

## Discussion

Our results indicate that the prevalence of diabetes is considerably high in the Mudanjiang area of China, especially in the Han population. The prevalences of diabetes in Manchu (8.39%) and Korean Chinese (9.42%) were slightly lower than the national average, while the prevalence of diabetes in Han Chinese in this area (12.10%) was higher than the prevalence in the overall Chinese population (9.7%) [7]. Prediabetes is an important risk factor for the development of overt diabetes and cardiovascular disease [8,9]. The prevalences of prediabetes identified in our study (18.96% in Han, 19.36% in Manchu and 20.47% in Korean Chinese populations) were also much higher than the overall prevalence in China (15.5%). These data have significant public health implications for estimating the burden of diabetes in the Mudanjiang area. The diagnosis of diabetes in the present study was based on both fasting plasma glucose levels and 2-h plasma glucose levels in an OGTT, and the measurement was performed in a qualified central lab. We believe our estimates of diabetes and prediabeteses are accurate.

According to our study, the prevalence of previously undiagnosed diabetes was significantly higher than previously diagnosed diabetes across the three ethnic groups. The proportions of undiagnosed diabetes were 69.94% in the Han population, 88.46% in the Manchu population and 90.48% in the Korean Chinese population, and were much higher than the overall rate in China (60.7%) [7]. These results raise concerns over the prevention and diagnosis of diabetes in this area, especially in minority groups. Though the prevalence of previously undiagnosed diabetes in the Han population is relatively low, ethnic Han are the major component of the Mudanjiang area population, and the absolute numbers of previously undiagnosed diabetes and prediabetes in the Han population were the largest. Healthcare professionals should work to increase the awareness of diabetes among residents. Active measures should be taken to screen for diabetes.

Diabetes was most frequent among the oldest age groups. Our data showed that the prevalence of diabetes rose with increasing age, and the relationship was steeper in the Han population. However, before being diagnosed with diabetes, a patient might have prediabetes for several years. It is well-known that diabetes can be prevented or delayed with early intervention [10-13]. Individuals with prediabetes are at increased risk of developing diabetes [14]. Therefore prediabetic individuals need attention for diabetes prevention and care. The prevalence of prediabetes among the three ethnic groups differed across age groups. The highest prevalence of prediabetes in the 20-40 year old group was observed in the Korean Chinese population, while in the 40-60 year old group and 60-80 year old group, Han and Manchu Chinese had the highest prevalence of prediabetes among the three ethnic populations, respectively. It is interesting to note that the prevalence of prediabetes is quite stable in Korean Chinese across the different age groups. We speculate that the differences in prediabetic characteristics could be partially attributed to ethnicity, and that this warrants further investigation.

Korean Chinese had higher prevalence of isolated IGT and lower prevalence of isolated IFG than the other two ethnic groups. People with IGT and IFG have different metabolic characteristics. It is reported that individuals with isolated IFG usually have impaired hepatorenal insulin sensitivity, while those with isolated IGT usually have impaired muscle insulin sensitivity [15]. In Meyer's study, individuals with isolated IGT showed impairments in basal insulin secretion and first-phase insulin release, whereas individuals with isolated IGT showed reduced second-phase insulin release and peripheral insulin resistance [16]. Further investigations are needed to examine β-cell function and insulin resistance among ethnic populations, which might explain the different profiles of prediabetes among the three ethnic groups.

In our study, the Manchu population had a lower risk of diabetes than the Han population. In the multivariate multinomial logistic regression, the Manchu ethnicity is still an independent protective factor against diabetes. The fact that the demographic and lifestyle factors tested could not explain all of the ethnic differences in diabetes prevalence indicates that other factors such as genetic susceptibility or other endogenous or environmental factors may be responsible for the lowest frequency of diabetes in the Manchu population.

The regression model showed that dietary control and weight monitoring were strongly associated with increased prevalence of diabetes and prediabetes. This might be because people usually begin to pay attention to their diet and body weight after the diagnosis of diabetes or obesity.

This study had several strengths. First, we are the first to present data of the current prevalence of diabetes and prediabetes among the three ethnic groups, Han, Manchu and Korean, in the Mudanjiang area of China. Second, the prevalences of diabetes and prediabetes were different among different age groups and different ethnic populations, and this finding provides fundamental information for the prevention and treatment of diabetes. Finally, that the Manchu ethnicity was a protective factor provides a clue for further investigation of genetic mechanisms.

In the present study, women residents were oversampled, and there was a lower response rate among men than among women. We took these issues into account when we calculated statistical weights.

## Conclusions

In summary, our results showed that the prevalence of diabetes varied among different ethnic populations, with a lower prevalence among Manchu Chinese. The Han population in the Mudanjiang area had a higher prevalence of diabetes than the national average. More troublesome is the finding that the majority of cases of diabetes were undiagnosed. These results of this study indicate that diabetes is a major public health challenge in the Mudanjiang area and active measures should be taken to prevent diabetes.

## Competing interests

The authors declare that they have no competing interests.

## Authors' contributions

YF and YY contributed equally to this work. JZ and QL are corresponding authors. YF, YY, PL, QL and JZ participated in the design of the study and performed the statistical analysis. YF, YY, XM, MW, KC, NW, DW conceived the study idea, participated in its design and coordination, and helped to draft the manuscript. All authors read and approved the final manuscript.

## Pre-publication history

The pre-publication history for this paper can be accessed here:

http://www.biomedcentral.com/1471-2458/12/23/prepub

## Supplementary Material

Additional file 1 **Characteristics of study participants by plasma glucose categories and ethnicity**. Click here for file
