# Supplementary material for: Prevalence of diabetes among Han, Manchu and Korean ethnicities in the Mudanjiang area of China: a cross-sectional survey
Source: BMC Public Health. 2012 Jan 10;12:23. doi: 10.1186/1471-2458-12-23 (PMC3298515; doi:10.1186/1471-2458-12-23)
Supplement: Additional file 1 — Characteristics of study participants by plasma glucose categories and ethnicity. [file 1471-2458-12-23-S1.DOC]

Additional file 1 Characteristics of study participants by plasma glucose categories and ethnicity

|  |  | Normal Glucose  Tolerance (n=2775) | Isolated Impaired  Fasting Glucose  (n=168) | Isolated Impaired  Glucose Tolerance  (n=404) | Combined Impaired Fasting  Glucose and Impaired  Glucose Tolerance  (n=196) | Previously  Undiagnosed  Diabetes  (n=331) | Previously  Diagnosed  Diabetes  (n=110) | Total  Prediabetes  (n=768) | Total  Diabetes  (n=441) |
| --- | --- | --- | --- | --- | --- | --- | --- | --- | --- |
| Age  (yr) | Han | 40.17±12.52 | 44.97±14.33 | 45.66±13.37 | 44.46±13.93 | 47.49±12.86 | 52.11±12.63 | 45.16±13.74 | 48.88±12.95 |
|  | Manchu | 40. 89±12.53 | 44.64±14.51 | 47.43±13.62 | 43.08±13.47 | 48.63±12.77 | 47.33±14.00 | 45.91±13.80 | 48.48±12.78 |
|  | Korean | 41.94±12.80 | 42.41±15.90^*^ | 41.84±12.76 | 43.37±12.94 | 45.77±13.85 | 44.67±11.06 | 42.25±13.13 | 45.67±13.54 |
| Family history  of diabetes (%) | Han | 28.15 | 33.33 | 30.49 | 39.44 | 32.46 | 57.14 | 33.66 | 39.88 |
|  | Manchu | 27.23 | 35.71 | 26.47 | 16.67 | 26.09* | 83.33* | 26.67* | 32.69* |
|  | Korean | 29.21 | 35.29 | 32.22 | 23.33 | 26.32* | 66.67* | 30.66* | 30.16* |
| Educational levels(%)  (senior high school or above) | Han | 31.11 | 26.83 | 24.80 | 32.39 | 32.02 | 31.63 | 27.40 | 31.90 |
|  | Manchu | 35.04 | 39.29 | 33.82 | 33.33 | 32.61 | 16.67 | 26.67 | 30.77 |
|  | Korean | 34.33 | 35.29 | 40.00 | 36.67 | 35.09 | 0 | 38.69* | 31.75 |
| Cigarette smoking (%) | Han | 27.99 | 27.64 | 26.01 | 32.39 | 22.36 | 34.69 | 26.42 | 26.07 |
|  | Manchu | 34.60 | 32.14 | 25.00 | 32.61 | 32.61* | 66.67* | 28.33 | 36.54* |
|  | Korean | 24.73 | 35.29 | 27.78 | 40.00 | 28.07* | 50.00* | 31.38* | 30.15* |
| Consumption of alcohol (%) | Han | 26.59 | 25.20 | 23.58 | 29.58 | 26.32 | 30.61 | 25.64 | 27.61 |
|  | Manchu | 24.78 | 28.57 | 29.41 | 41.67 | 21.74 | 33.33* | 29.17* | 23.08* |
|  | Korean | 25.80 | 52.94 | 27.78 | 30.00 | 19.30* | 16.67* | 31.39* | 19.05* |
| Level of economic (%)  (10000 >yuanl/yr) | Han | 35.23 | 39.84 | 36.59 | 35.21 | 36.40 | 47.96 | 36.99 | 39.88 |
|  | Manchu | 39.29 | 35.71 | 45.59 | 54.17 | 39.13 | 66.67 | 45.00* | 42.31* |
|  | Korean | 37.95 | 29.41 | 36.67 | 33.33 | 36.84 | 66.67 | 35.04 | 39.68 |
| Physical activity (%) | Han | 29.12 | 30.08 | 27.23 | 28.17 | 24.56 | 42.86 | 28.18 | 30.06 |
|  | Manchu | 24.11 | 10.71 | 27.94 | 12.50 | 39.13* | 50.00* | 20.83* | 40.38* |
|  | Korean | 30.49 | 47.06 | 23.33 | 33.33 | 22.81 | 50.00* | 28.47 | 25.40* |
| FPG  (mmol/L) | Han | 5.25±0.51 | 6.37±0.21 | 5.50±0.42 | 6.49±0.23 | 8.44±2.10 | 9.10±2.64 | 5.98±0.57 | 8.64±2.29 |
|  | Manchu | 5.20±0.51 | 6.40±0.24 | 5.56±0.34 | 6.41±0.23 | 8.30±2.26* | 7.28±0.44* | 5.92±0.52 | 8.18±2.15 |
|  | Korean | 5.26±0.47 | 6.35±0.24 | 5.55±0.37 | 6.54±0.26 | 9.69±3.00* | 11.04±3.96* | 5.87±0.55 | 9.81±3.09 |
| 2-hr plasma  glucose in OGTT  (mmol/L) | Han | 5.79±1.15 | 6.54±0.97 | 8.88±0.86 | 9.09±0.80 | 14.78±4.41 | 16.92±4.04 | 8.38±1.36 | 15.43±4.41 |
|  | Manchu | 5.91±1.19 | 6.78±0.78 | 8.80±0.81 | 9.43±0.93 | 15.77±3.76 | 15.55±2.62 | 8.45±1.26 | 15.74±3.63 |
|  | Korean | 5.85±1.09 | 6.55±1.16 | 8.80±0.82 | 9.28±0.97 | 17.00±4.58* | 19.55±6.33* | 8.63±1.21 | 17.24±4.77* |
| BMI  (kg/m^2^) | Han | 25.21±3.88 | 26.46±4.56 | 25.95±4.00 | 26.10±3.14 | 26.00±4.15 | 26.57±3.58 | 26.11±3.93 | 26.17±3.99 |
|  | Manchu | 25.14±3.16 | 26.32±2.76 | 26.27±2.87 | 26.50±3.64 | 26.87±2.32 | 26.16±2.17 | 26.33±2.99 | 26.79±2.29 |
|  | Korean | 25.21±2.99 | 25.42±2.69 | 25.58±2.97 | 25.63±2.74 | 26.55±5.23 | 26.17±2.11 | 25.57±2.87 | 26.51±5.01 |
| Waist circumference  (cm) | Han | 77.94±9.36 | 81.37±10.32 | 79.98±9.90 | 82.20±9.55 | 80.26±8.65 | 85.47±10.65 | 80.93±9.94 | 81.83±9.58 |
|  | Manchu | 76.82±3.16 | 81.41±8.07 | 80.27±7.87 | 82.06±6.12 | 80.58±7.95 | 79.95±9.98 | 80.90±7.58 | 80.51±8.10 |
|  | Korean | 77.88±8.14 | 78.62±7.72 | 79.56±8.52 | 80.23±8.51 | 80.39±8.17 | 76.75±4.52 | 79.59±8.38 | 80.05±7.95 |
| WHR | Han | 0.80±0.08 | 0.82±0.08 | 0.85±0.45 | 0.83±0.07 | 0.81±0.08 | 0.85±0.08 | 0.84±0.31 | 0.83±0.08 |
|  | Manchu | 0.80±0.07 | 0.83±0.07 | 0.81±0.08 | 0.83±0.05 | 0.80±0.05 | 0.82±0.10 | 0.82±0.07* | 0.81±0.06* |
|  | Korean | 0.80±0.07 | 0.78±0.07 | 0.81±0.07 | 0.80±0.07 | 0.81±0.08 | 0.78±0.04* | 0.80±0.07* | 0.81±0.07* |
| SBP  (mmHg) | Han | 126.22±19.56 | 129.98±19.78 | 129.20±18.60 | 129.69±19.55 | 132.74±19.35 | 140.72±19.10 | 129.52±19.11 | 135.14±19.59 |
|  | Manchu | 122.33±17.30* | 128.11±16.37 | 135.13±22.69 | 127.17±15.93 | 140.70±19.30* | 127.33±18.95* | 131.90±20.33 | 139.15±19.56 |
|  | Korean | 122.80±19.37* | 127.06±19.37 | 126.97±17.09* | 132.33±19.50 | 136.91±17.92* | 135.17±22.95* | 128.15±17.92 | 136.75±18.24 |
| Resting heart rate  (beat/min) | Han | 76.44±9.07 | 75.68±8.78 | 77.38±9.14 | 77.87±11.07 | 78.74±9.56 | 79.74±10.82 | 77.11±9.65 | 79.04±9.95 |
|  | Manchu | 76.86±8.88 | 75.57±8.21 | 77.96±12.02 | 79.96±10.32 | 80.43±9.88 | 74.66±5.16 | 77.80±10.92 | 79.77±9.61 |
|  | Korean | 76.67±8.57 | 76.12±7.86 | 75.71±8.71 | 79.33±13.00 | 77.84±9.97 | 85.83±4.58 | 76.55±9.75 | 78.60±9.85 |
| HDL-C  (mmol/L) | Han | 1.45±0.38 | 1.44±0.46 | 1.38±0.39 | 1.43±0.46 | 1.43±0.41 | 1.33±0.36 | 1.41±0.43 | 1.40±0.40 |
|  | Manchu | 1.48±0.37 | 1.45±0.35 | 1.38±0.40 | 1.51±0.57 | 1.36±0.32 | 1.25±0.20 | 1.43±0.43 | 1.35±0.31 |
|  | Korean | 1.45±0.41 | 1.48±0.54 | 1.43±0.47 | 1.49±0.46 | 1.32±0.46 | 1.29±0.22 | 1.45±0.48 | 1.32±0.44 |
| LDL-C  (mmol/L) | Han | 2.61±0.81 | 2.72±0.93 | 2.65±0.82 | 2.75±0.90 | 2.92±0.97 | 2.79±1.09 | 2.69±0.87 | 2.88±1.01 |
|  | Manchu | 2.59±0.75 | 2.90±0.80 | 2.90±1.06 | 3.06±1.39 | 2.67±0.82 | 2.56±0.79 | 2.93±1.07 | 2.66±0.81 |
|  | Korean | 2.60±0.79 | 2.77±1.13 | 2.63±0.74 | 2.73±1.17 | 2.83±1.06 | 2.80±0.59 | 2.67±0.89 | 2.83±1.02 |
| TG  (mmol/L) | Han | 1.63±1.49 | 2.04±2.36 | 2.07±1.95 | 1.95±1.14 | 2.27±2.63 | 2.88±4.19 | 2.03±1.88 | 2.45±3.19 |
|  | Manchu | 1.59±1.25 | 1.83±0.62 | 2.46±4.30 | 1.83±0.78 | 2.31±1.82 | 1.47±0.72 | 2.19±3.27 | 2.22±1.75 |
|  | Korean | 1.62±1.64 | 1.86±1.38 | 1.82±1.16 | 1.89±0.93 | 3.05±2.70 | 3.91±2.05 | 1.85±1.14 | 3.13±2.64 |

All data, including percentages and mean values, were weighted to represent the total population of Mudanjiang area adults (aged 20 years or older) on the basis of Mudanjiang population data from 2000. Cigarette smoking was defined as having smoked at least 100 cigarettes in one’s lifetime. Alcohol drinking was defined as consumption of at least 30 g of alcohol per week for 1 year or more. Regular leisure-time physical activity was defined as participation in 30 minutes or more of moderate or vigorous activity per day, at least 3 days per week. Body-mass index is weight in kilograms divided by the square of height in meters.

*p<0.05 vs Han.
